# How Commonly Is the Diagnosis of Gastric Low Grade Dysplasia Upgraded following Endoscopic Resection? A Meta-Analysis

**DOI:** 10.1371/journal.pone.0132699

**Published:** 2015-07-16

**Authors:** Guangfeng Zhao, Meng Xue, Yingying Hu, Sanchuan Lai, Shujie Chen, Liangjing Wang

**Affiliations:** 1 Department of Emergency, the Second Affiliated Hospital, School of Medicine, Zhejiang University, Hangzhou, Zhejiang, China; 2 Department of Gastroenterology, the Second Affiliated Hospital, School of Medicine, Zhejiang University, Hangzhou, Zhejiang, China; 3 Institute of Gastroenterology, Zhejiang University, Hangzhou, Zhejiang, China; 4 Department of Gastroenterology, Sir Runrun Shaw Hospital, School of Medicine, Zhejiang University, Hangzhou, Zhejiang, China; Shanghai Jiao Tong University School of Medicine, CHINA

## Abstract

Gastric dysplasia is a well-known precancerous lesion. Though the diagnosis of gastric low grade dysplasia (LGD) is generally made from endoscopic forceps biopsy (EFB), the accuracy is doubtful after numerous EFB-proven gastric LGD were upgraded to gastric high grade dysplasia (HGD) or even carcinoma (CA) by further diagnostic test with the procedure of endoscopic resection (ER). We aimed to evaluate the upgraded diagnosis rate (UDR) and the risk factors by ER in EFB-proven gastric LGD lesions. Two investigators independently searched studies reporting the UDR by ER in EFB-proven gastric LGD lesions from databases and analyzed the overall UDR, HGD-UDR and CA-UDR. The pooled UDR by ER in EFB-proven gastric LGD lesions was 25.0% (95% CI, 20.2%-29.8%), made up of HGD-UDR and CA-UDR by rates of 16.7% (95% CI, 12.8%-20.6%) and 6.9% (95% CI, 4.2%-9.6%) respectively. Lesion size larger than 2 cm, surface with depression and nodularity under endoscopic examinations were the major risk factors associated with UDR. In conclusion, one quarter of EFB-proven gastric LGD lesions will be diagnosed as advanced lesions, including gastric HGD (16.7%) and gastric CA (6.9%) by ER. The diagnosis of those LGD lesions with an endoscopic diameter larger than 2cm, and depressed or nodular surface are more likely to be upgraded after ER.

## Introduction

Gastric dysplasia, or named as gastric epithelial neoplasia, is a critical step in the gastric precancerous cascade [1], characterized by cellular atypia, abnormal differentiation and disorganized mucosal architecture [2]. Up to 7.3% of those patients receiving gastroscopy examinations would be diagnosed as gastric dysplasia in Asian countries such as China [3], much higher than that in the Western world [4]. In spite of its noninvasive nature [5], gastric dysplasia lesion has been brought to the forefront due to its risk progressing to gastric cancer [6].

Gastric dysplasia could be divided into different pathological types, including adenomatous dysplasia, foveolar dysplasia, tubular neck dysplasia and polypoid gastric dysplasia (or gastric adenoma) [7]. Before the widespread use of endoscopic resection (ER) and a unified grading system of gastric dysplasia, gastroenterologists might face a dilemma whether suggesting patients with gastric dysplasia or epithelial neoplasia to gastrectomy or not. Due to different nomenclature and grading systems, diagnosis discrepancy exists between Western and Eastern pathologists. The Vienna meeting in 1998 established a grading system, in which gastrointestinal epithelial neoplasia were grouped into 5 categories. Non-invasive low grade adenoma/dysplasia come under category 3, while non-invasive high grade adenoma/dysplasia come under category 4 [8].

High grade dysplasia (HGD) has a 75% risk associating with or progressing to carcinoma (CA), so there is no doubt that gastric HGD is a precancerous lesion of gastric CA [9] and local ER, including endoscopic resection (EMR) and endoscopic submucosal dissection (ESD), should be recommended as further treatment [10,11]. However, the clinical criteria for the management of gastric low grade dysplasia (LGD) were not clear [10]. Gastric LGD patients have a relative lower risk progressing to CA [12,13]. In addition, ER would carry a risk of complications including gastric bleeding and perforation [14], increase the cost and require hospital admission [15]. With regards to the clinical challenge of LGD, the management of precancerous conditions and lesions in the stomach (MAPS) guidelines stated that ER should be considered only in patients with endoscopically defined lesions in order to obtain a more accurate histological diagnosis, otherwise patients with LGD could receive follow-up annually after diagnosis [16].

The initial solution to obtain gastric mucosa tissues is endoscopic forceps biopsy (EFB). While subject to the limitations of superficial and inadequate tissues, plus the multifocal nature of these lesions, EFB will be inevitably accompanied with false negative [17]. A series of studies have reported that diagnosis of gastric LGD by EFB would be upgraded to gastric HGD or even CA after ER [4,15,18–31]. However, the upgraded diagnosis rate (UDR) seems largely discrepant among these studies, ranging from 10.0% to 46.7%. The aim of this meta-analysis is to evaluate the UDR by ER in EFB-proven gastric LGD lesions and the possible risk factors associated with UDR systematically.

## Materials and Methods

### Data identification and study selection

Databases PubMed, Medline, Web of science, Embase, Scopus, Ovid and the Cochrane Library were searched with the following terms: (gastric epithelial neoplasia OR gastric dysplasia) AND biopsy AND (endoscopic resection OR Endoscopic submucosal dissection OR Endoscopic mucosal resection). Publications from January, 2000 to March, 2014 were searched by two independent investigators. Studies were required to fulfill the following inclusion criteria: (1) written in English; (2) lesions of gastric LGD were initially diagnosed by EFB and the total number was available; (3) post-ER pathology for these LGD lesions were recorded separately. As the procedure of endoscopic resection is similar with EMR, data of cap-assisted EMR and endoscopic snare polypectomy in two studies were integrated into EMR [4,31].

Primary screening was based on publication type, language, title and abstract. Reviews (consensus, guidelines and systematic review included), case reports or case series, not written in English or lesions other than gastric LGD were excluded. In terms of the remaining ones, without reporting post-ER pathological results for gastric LGD, or only reported post-ER pathological results without initial biopsy results, or having no separate analysis of gastric LGD were ruled out. To obtain reliable results, any study with a sample size smaller than 10 was not taken into account in the final analysis [32, 33]. If the results of 2 or more studies were based on a repeated patient cohort, then only the newest one was included. Different opinions between authors were resolved by discussion.

### Data extraction

Two authors extracted data from each selected study including the following items: (1) first author’s name and the year of publication; (2) design of the study (prospective or retrospective); (3) whether consecutive patients were included; (4) the country where the study was conducted; (5) whether it was a single-center or multi-center study; (6) publication type (full text or abstract); (7) which kind of ER methods was applied (ESD or EMR); (8) the number of gastric LGD lesions, and the number of patients with gastric LGD diagnosed by EFB (if available); (9) the number of gastric LGD lesions upgrading to HGD diagnosed by ER (for HGD-UDR); (10) the number of gastric LGD lesions upgrading to carcinoma diagnosed by ER (for CA-UDR); (11) the number of gastric LGD lesions upgrading to HGD or carcinoma diagnosed by ER (for UDR); (12) male/female ratio, the average age (age range) of the gastric LGD patients (if available).

### Risk of bias in individual studies

The quality of included studies was assessed by the Quality Assessment of Diagnostic Accuracy Studies (QUADAS). Each item was marked as “yes” if the criteria was met, as”no” if not met, and “unclear” if the information supplied was not sufficient.

### Statistical analysis

Data of UDR, HGD-UDR and CA-UDR were calculated, pooled, and analyzed. Q statistic of *I* ^*2*^ was used to estimate the proportion of unexplained variation across studies. *I* ^*2*^ >50% was considered significant for heterogeneity, which would indicate the use of the random-effect model, DerSimonian-Laird method to derive pooled results with corresponding 95% CI.

Meta-regression analysis was used to investigate the possible sources of heterogeneity based on the following covariates: publication type (full text vs abstract only), number of centers (multi vs single), design of study (prospective vs retrospective), and sample size (>60 lesions vs ≤60 lesions). Publication bias was assessed using funnel plots (based on UDR versus the standard error). Statistical analysis was carried out using the Metan, Metareg and Metabias packages of STATA version 12.0 (StataCorp, College Station, Tex).

## Results

### Description of studies

**Fig 1:**
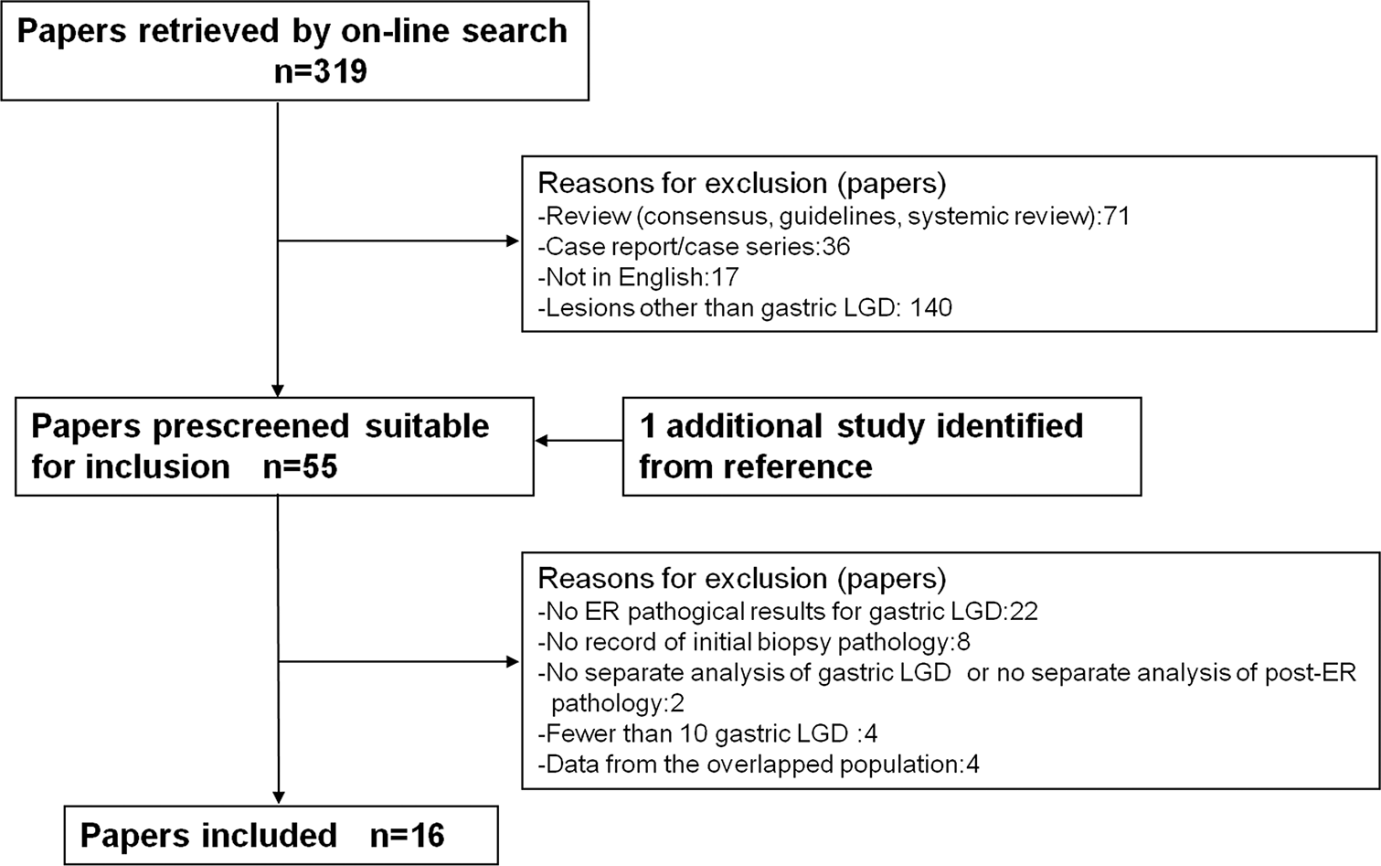
Flow chart of the process for selecting eligible studies. LGD, low grade dysplasia; ER, endoscopic resection.

A total of 319 papers were retrieved after initial search and 1 additional study was identified from reference. According to the aforementioned inclusion and exclusion criteria, 16 papers were screened out eligible for further analysis. A flow chart in **Fig 1** demonstrated the process for selecting eligible studies. Those 16 studies reported 3033 EFB-proven gastric LGD lesions along with documented post-ER pathology.

The main characteristics of the eligible studies are presented in **Table 1**. Most of the studies were conducted in Korea [4,15,18–23,25,27–29,31], except for 1 in Japan [24], 1 in Japan and the United States [30] and 1 in Italy [26]. Full texts were available for 13 of the included studies, and the remaining 3 studies were meeting abstracts. Three of these included studies were prospectively designed. Single ESD was applied in 3 of the studies, single EMR in 4 of the studies, and either ESD or EMR in the remaining 9 studies. UDR in gastric LGD lesions ranged from 10.0% (3/30) [26] to 46.7% (64/137) [24], with HGD-UDR from 6.7% (2/30) [26] to 30.8% (4/13) [30], and CA-UDR from 1.0% (1/96) [31] to 15.5% (39/251) [25], respectively.

**Table 1:** Characteristics of the eligible studies.

	Design	Consecu.	Country	Number of centers	Publication type	ER methods	Number of LGD(EFB) patients	Number of LGD(EFB) lesions	Gender, male/female	Age Mean (Range)	Number of HGD(ER)	Number of CA(ER)	Number of HC(ER)
Choi 2014^[^ ^18^ ^]^	Pro.	Yes	Korea	Single	Full	ESD	NA	218	151/67^‡^	62^‡^	NA	NA	38
Kim 2014^[^ ^19^ ^]^	Retro.	Yes	Korea	Single	Full	ESD/EMR	257	285	201/84^‡^	63.8^‡^	22	24	46
Lim 2014^[^ ^20^ ^]^	Retro.	Yes	Korea	Single	Full	ESD/EMR	NA	954	NA	NA	114	115	229
Jeon 2013^[^ ^21^ ^]^	Pro.	Yes	Korea	Single	Full	ESD	NA	54	NA	NA	9	3	12
Kim 2012^[^ ^22^ ^]^	Retro.	Yes	Korea	Single	Full	ESD/EMR	99	NA	NA	NA	15	11	26
Hwang 2012^[^ ^23^ ^]^	Retro.	Yes	Korea	Single	Abstract	ESD/EMR	NA	70	NA	NA	6	2	8
Tsuji 2012^[^ ^24^ ^]^	Retro.	Yes	Japan	Single	Full	ESD	NA	137	101/36^‡^	67.7^‡^	NA	NA	64
Cho 2011^[^ ^15^ ^]^	Retro.	Yes	Korea	Single	Full	ESD/EMR	208	236	174/62^‡^	61.6^‡^	71	9	80
Won 2011^[^ ^25^ ^]^	Retro.	Yes	Korea	Single	Full	ESD/EMR	241	251	175/66^†^	62.6^†^	56	39	95
Suriani 2011^[^ ^26^ ^]^	Pro.	Yes	Italy	Multi	Abstract	EMR	30	30	NA	NA	2	1	3
Lee 2010^[^ ^27^ ^]^	Retro.	Yes	Korea	Single	Full	ESD/EMR	NA	208	NA	NA	54	16	70
Kim 2010^[^ ^4^ ^]^	Retro.	Yes	Korea	Single	Full	ESD/EMR	273	273	181/92	62.9(35–87)	27	24	51
Sung 2009^[^ ^28^ ^]^	Retro.	Yes	Korea	Single	Full	ESD/EMR	NA	55	NA	NA	16	4	20
Kim 2006^[^ ^29^ ^]^	Retro.	Yes	Korea	Single	Abstract	EMR	54	54	NA	NA	13	1	14
Lauwers 2004^[^ ^30^ ^]^	Retro.	Yes	Japan & USA	Multi	Full	EMR	13	13	NA	NA	4	1	5
Park 2001^[^ ^31^ ^]^	Retro.	Yes	Korea	Single	Full	EMR	96	96	NA	NA	12	1	13

Pro., Prospective; Retro., Retrospective; Consecu., Consecutive; ER, endoscopic resection; EMR: endoscopic mucosal resection; ESD: endoscopic submucosal dissection; LGD, low grade dysplasia; EFB, endoscopic forceps biopsy; HGD, high grade dysplasia; ER, endoscopic resection; CA, carcinoma; HC, high grade dysplasia plus carcinoma;

^†^Gender and Age are counted based on number of LGD (EFB) patients

^‡^Gender and Age are counted based on number of LGD (EFB) lesions.

### Quality of studies

The quality of each selected study was evaluated with 14 items using the QUADAS tool (**Table 2**). In the studies conducted by Kim et al [4] and Choi et al [18], ER was performed 349 days and 3 years later than the initial EFB in part of the subjects, so the time interval between ER and EFB wasn’t short enough to make sure that the lesion wouldn’t change during this period of time, accordingly, item 4 was marked as “No”; In another two studies [19,21], ER was performed within 1 month and marked as “yes”; Fourteen studies didn’t report the time interval, so item 4 was marked as “unclear”. In terms of item 11, none of the included studies reported whether the endoscopists or pathologists were aware of the EFB results when they performed the ER or read the slides. As a result, all included studies were marked as “unclear” on this item. All the included studies met the criteria of the remaining 12 items, suggesting a good quality for most of the studies.

**Table 2:** Quality of studies using QUADAS tool.

	Item1	Item2	Item3	Item4	Item5	Item6	Item7	Item8	Item9	Item10	Item11	Item12	Item13	Item14
Choi 2014^[^ ^18^ ^]^	Y	Y	Y	N	Y	Y	Y	Y	Y	Y	U	Y	Y	Y
Kim 2014^[^ ^19^ ^]^	Y	Y	Y	Y	Y	Y	Y	Y	Y	Y	U	Y	Y	Y
Lim 2014^[^ ^20^ ^]^	Y	Y	Y	U	Y	Y	Y	Y	Y	Y	U	Y	Y	Y
Jeon 2013^[^ ^21^ ^]^	Y	Y	Y	Y	Y	Y	Y	Y	Y	Y	U	Y	Y	Y
Kim 2012^[^ ^22^ ^]^	Y	Y	Y	U	Y	Y	Y	Y	Y	Y	U	Y	Y	Y
Hwang 2012^[^ ^23^ ^]^	Y	Y	Y	U	Y	Y	Y	Y	Y	Y	U	Y	Y	Y
Tsuji 2012^[^ ^24^ ^]^	Y	Y	Y	U	Y	Y	Y	Y	Y	Y	U	Y	Y	Y
Cho 2011^[^ ^15^ ^]^	Y	Y	Y	U	Y	Y	Y	Y	Y	Y	U	Y	Y	Y
Won 2011^[^ ^25^ ^]^	Y	Y	Y	U	Y	Y	Y	Y	Y	Y	U	Y	Y	Y
Suriani 2011^[^ ^26^ ^]^	Y	Y	Y	U	Y	Y	Y	Y	Y	Y	U	Y	Y	Y
Lee 2010^[^ ^27^ ^]^	Y	Y	Y	U	Y	Y	Y	Y	Y	Y	U	Y	Y	Y
Kim 2010^[^ ^4^ ^]^	Y	Y	Y	N	Y	Y	Y	Y	Y	Y	U	Y	Y	Y
Sung 2009^[^ ^28^ ^]^	Y	Y	Y	U	Y	Y	Y	Y	Y	Y	U	Y	Y	Y
Kim 2006^[^ ^29^ ^]^	Y	Y	Y	U	Y	Y	Y	Y	Y	Y	U	Y	Y	Y
Lauwers 2004^[^ ^30^ ^]^	Y	Y	Y	U	Y	Y	Y	Y	Y	Y	U	Y	Y	Y
Park 2001^[^ ^31^ ^]^	Y	Y	Y	U	Y	Y	Y	Y	Y	Y	U	Y	Y	Y

QUADAS, Quality Assessment of Diagnostic Accuracy Studies; Y, yes; N, no; U, unclear. Item 1. Was the spectrum of patients representative of the patients who will receive the test in practice? Item 2. Were selection criteria clearly described? Item 3. Is the reference standard likely to correctly classify the target condition? Item 4. Is the time period between reference standard and index test short enough to be reasonably sure that the target condition did not change between the two tests? Item 5. Did the whole sample or a random selection of the sample receive verification by using a reference standard of diagnosis? Item 6. Did patients receive the same reference standard regardless of the index test result? Item 7. Was the reference standard independent of the index test? Item 8. Was the execution of the index test described in sufficient detail to permit replication of the test? Item 9. Was the execution of the reference standard described in sufficient detail to permit its replication? Item 10. Were the index test results interpreted without knowledge of the results of the reference standard? Item 11. Were the reference standard results interpreted without knowledge of the results of the index test? Item 12. Were the same clinical data available when test results were interpreted as would be available when the test is used in practice? Item 13. Were uninterpretable/intermediate test results reported? Item 14. Were withdrawals from the study explained?

### Upgraded diagnosis rate of gastric LGD

**Fig 2:**
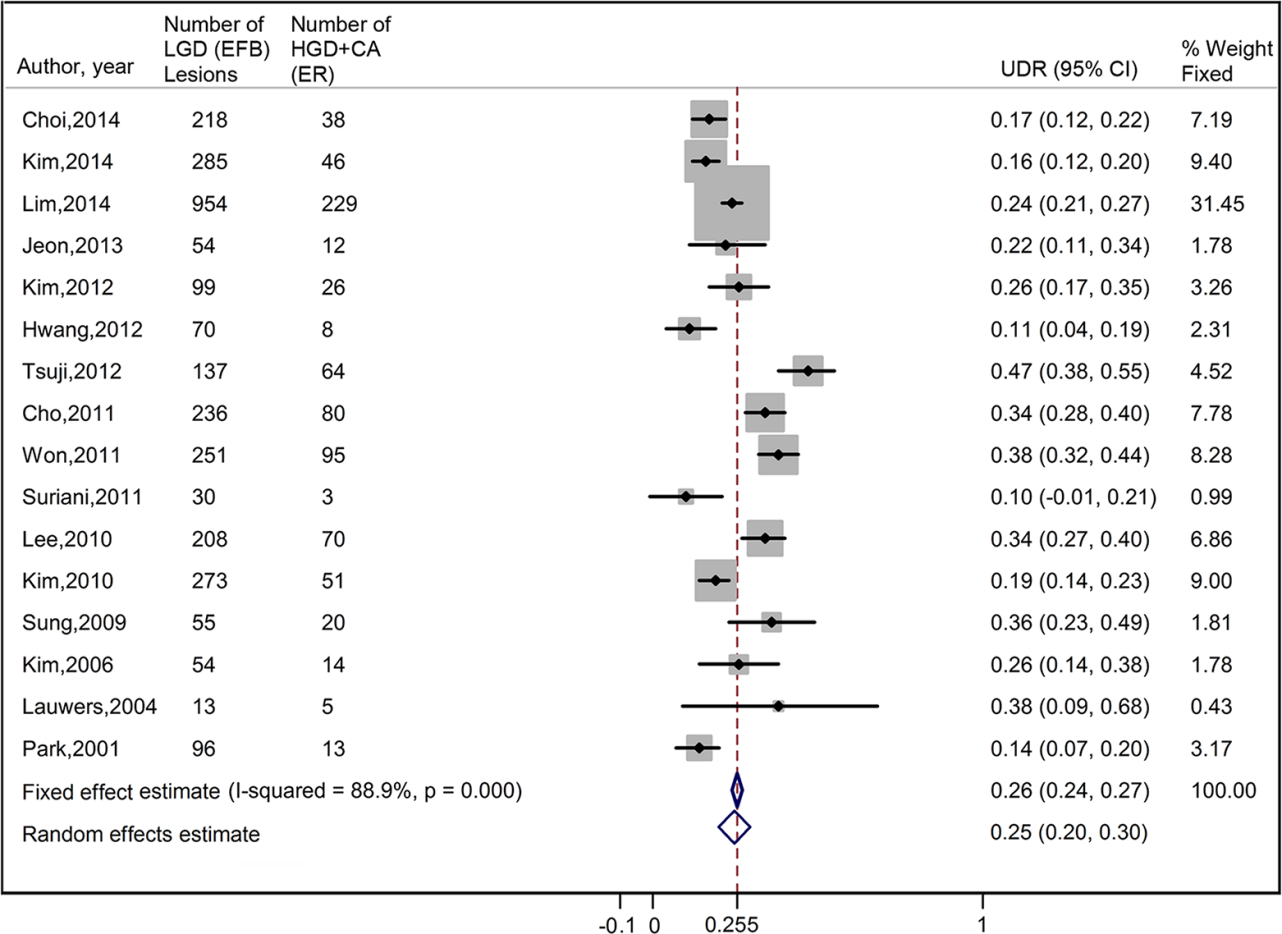
Forrest Plot of the overall UDR by ER in EFB-proven gastric LGD lesions. LGD, low grade dysplasia; EFB, endoscopic forceps biopsy; ER, endoscopic resection; UDR, upgraded diagnosis rate; CI, confidence interval.

**Fig 3:**
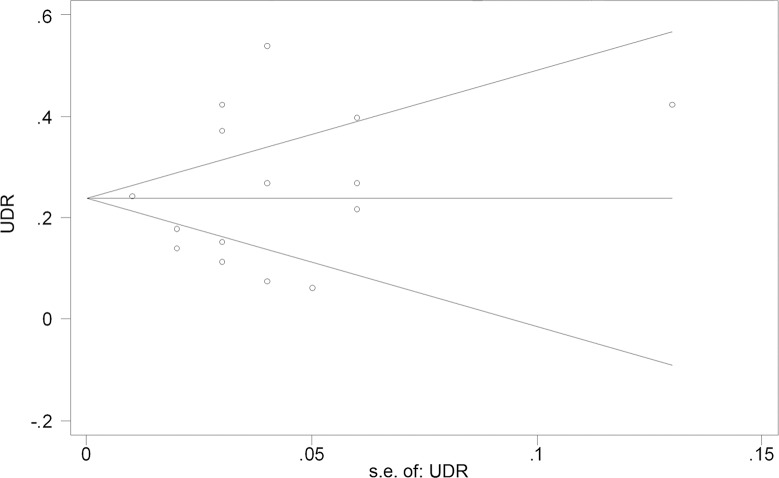
Funnel plot comparing overall UDR vs. standard error (Begg’s asymmetry test). UDR, upgraded diagnosis rate; s.e., standard error;

The pooled UDR in gastric LGD lesions, calculated by a random effects model, was 25.0% (95% CI, 20.2%-29.8%) (**Fig 2**). Significant heterogeneities existed among the eligible studies (*I* ^*2*^ = 88.9%, *p*<0.001). Multivariable meta-regression analysis showed that none of the publication type (*p* = 0.087), number of centers (*p* = 0.981), design of the studies (*p* = 0.117), or sample sizes (*p* = 0.239) was the source of heterogeneity. Begg’s funnel plot of UDR versus standard error showed symmetry (*p* for bias = 0.551) (**Fig 3**), indicating that there was no significant publication bias regarding to UDR in this systematic review.

**Fig 4:**
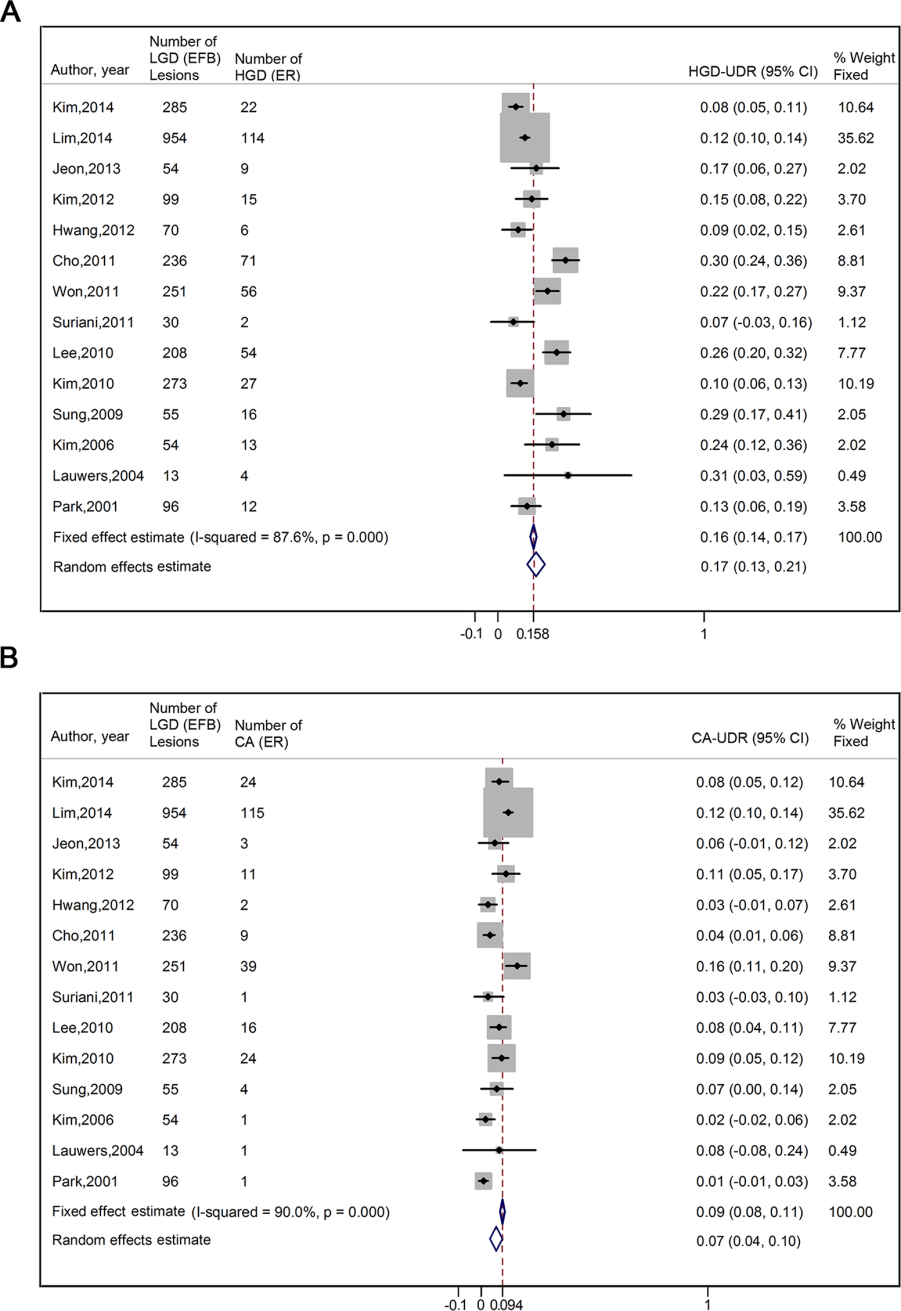
Forrest Plot of the type-specific UDR by ER in EFB-proven gastric LGD lesions. (A) HGD-UDR, (B) CA-UDR. LGD, low grade dysplasia; EFB, endoscopic forceps biopsy; HGD, high grade dysplasia; CA, carcinoma; ER, endoscopic resection; UDR, upgraded diagnosis rate; CI, confidence interval.

**Fig 5:**
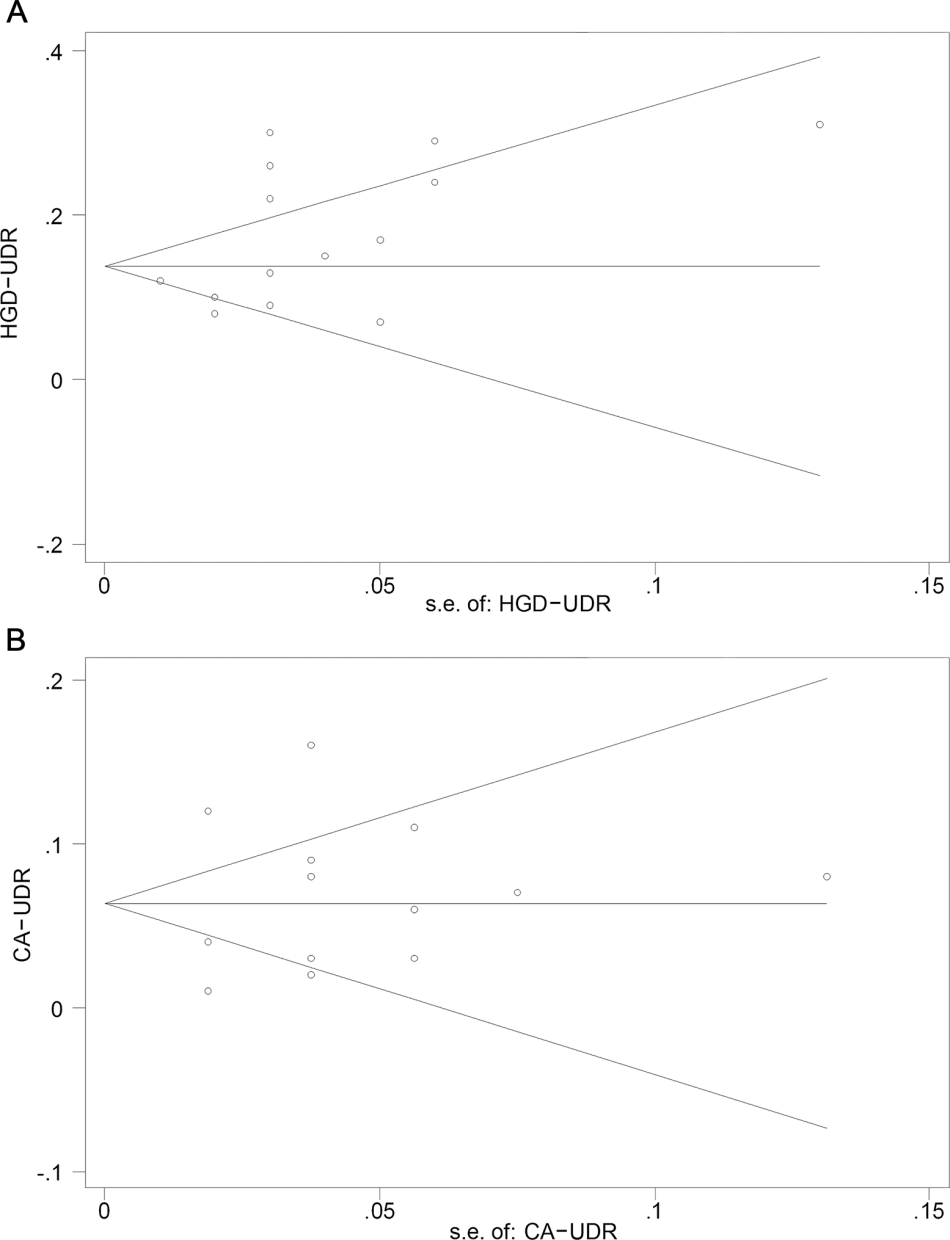
Funnel plot comparing type-specific UDR vs. standard error of UDR (Begg’s asymmetry test). (A) HGD-UDR, (B) CA-UDR. UDR, upgraded diagnosis rate; s.e., standard error; HGD, high grade dysplasia; CA, carcinoma.

Fourteen studies [4,15,19–23,25–31] reported the specific upgraded diagnosis results, enabling us to analyze HGD-UDR and CA-UDR separately. The pooled HGD-UDR and CA-UDR were 16.7% (95% CI, 12.8%-20.6%) and 6.9% (95% CI, 4.2%-9.6%), respectively (**Fig 4**). Significant heterogeneities also existed among the eligible studies (*I* ^*2*^ = 87.6% for HGD and 90.0% for CA, p<0.001 for both). Begg’s funnel plot showed symmetry for both of them (*p* for bias of HGD = 0.084, and of CA = 0.628) (**Fig 5**), indicating that there was no significant publication bias regarding to HGD-UDR and CA-UDR.

Given that excluding certain outliers might reduce the heterogeneities [34], we deleted 1 to 4 out of 4 possible outliers (two maximums and two minimums) and calculated the consequent *I* ^*2*^. Unfortunately, no obvious reduction of *I* ^*2*^ was observed for UDR and HGD-UDR, and the consequent *I* ^*2*^ for CA-UDR would fall below 50% only when all these 4 studies were excluded at the same time (**S1–S3 Tables**).

Eight studies [4,15,18–20,24,25,29] reported the risk factors of EFB-proven gastric LGD upgrading to HGD or CA. Among them, 100% (8/8) stated that lesion size was an independent factor indicating advanced histology of resected specimens in LGD by first biopsy. Six studies reported “cut-off” size suggesting advanced lesions, 2cm was a common diameter associated with a higher risk. Besides, depressed morphology, surface unevenness (or nodularity), erythema (or redness) and erosion were significant risk factors indicating post-ER upgraded diagnosis in gastric category 3 lesions, reported by 75% (6/8), 67% (4/6), 57% (4/7) and 25% (1/4) of these eight studies, respectively (**Table 3**).

**Table 3:** Risk factors indicating advanced histology of resected specimen in LGD.

		Surface			
Author, Year	Size(mm)	Erythema or redness	Unevenness or Nodularity	Erosion	Depressed gross
Choi 2014^[^ ^18^ ^]^	●(10)	●	●	○	○
Kim 2014^[^ ^19^ ^]^	●(20)	●	○	⊙	●
Lim 2014^[^ ^20^ ^]^	●	○	●	NA	●
Jeon 2013^[^ ^21^ ^]^	NSA	NSA	NSA	NSA	NSA
Kim 2012^[^ ^22^ ^]^	NA	NA	NA	NA	NA
Hwang 2012^[^ ^23^ ^]^	NA	NA	NA	NA	NA
Tsuji 2012^[^ ^24^ ^]^	⊙	⊙	NA	NA	⊙
Cho 2011^[^ ^15^ ^]^	●(10)	●	⊙	○	●
Won 2011^[^ ^25^ ^]^	●(15)	○	⊙	NA	⊙
Suriani 2011^[^ ^26^ ^]^	NA	NA	NA	NA	NA
Lee 2010^[^ ^27^ ^]^	NSA	NSA	NSA	NSA	NSA
Kim 2010^[^ ^4^ ^]^	⊙(10–30)^†^	NA	NA	NA	○
Sung 2009^[^ ^28^ ^]^	NA	NA	NA	NA	NA
Kim 2006^[^ ^29^ ^]^	●(15)	○	○	○	●
Lauwers 2004^[^ ^30^ ^]^	NA	NA	NA	NA	NA
Park 2001^[^ ^31^ ^]^	NA	NA	NA	NA	NA

LGD, low grade dysplasia; ●, Association proven by multivariate analysis or logistic regression analysis; ⊙, Association only proven by univariate analysis, not proven by multivariate analysis or logistic regression analysis; ○, No association proven by univariate or multivariate analysis; NA, No analysis; NSA, No separate analysis, only the total discrepancy rate (including both upgrading and downgrading) between EFB and ER specimens was recorded. ^†^The difference is significant no matter 10mm, 20mm or 30mm was set as “cut-off”.

Downgraded diagnosis rates were reported in 6 studies [20,25–29]. Among a total of 1552 lesions of EFB-proven gastric LGD, 99 (6.4%) were confirmed as nothing neoplastic but nonspecific chronic gastritis on histological examination after ER. Unfortunately, risk factors for downgraded diagnosis on ER specimens were not available from these studies.

## Discussion

Long-term follow-up studies showed that less than 10% of gastric LGD would progress to CA [12,13]. In addition, further ER might be associated with procedure complications [14] and would increase medical cost [35]. So in most situations, only endoscopic surveillance was recommended for gastric LGD lesions after the initial diagnosis. However, a considerable amount of recent literatures reported missing advanced findings by EFB, which might steal the opportunity of ER away from patients until it’s too late when the lesion has already progressed to invasive gastric CA [36]. To our best knowledge, this is the first meta-analysis focusing on the misdiagnosis rate by EFB alone in gastric LGD lesions. The pooled results indicate that in 3303 patients with EFB-proven gastric LGD lesions, 1 out of 4 might be misdiagnosed and should actually be HGD or even gastric CA. The missing rate is surprisingly high, reminding us that a “wait and see” approach is far from enough for those high-risk patients. It is meaningful to find out which kind of gastric LGD is likely to be misdiagnosed, so that further target biopsy or ER would be performed timely.

Our review collected the data from 8 studies reporting the risk factors associated with upgraded diagnosis by ER in EFB-proven LGD patients. All those studies mentioned that the larger the LGD lesion was, the more likely it would be an advanced lesion. In addition to lesion size, several studies also reported that surface depression, unevenness, redness and erosion were associated with the discrepancy between the pathological diagnosis by EFB and ER. This association gave us a hint when further target biopsy such as magnifying endoscopy with narrow-band imaging (ME-NBI), extra special biopsy including the jumbo biopsy and multisite biopsy, or ER should be recommended.

ME-NBI provides a resolution smaller than the minimal diameter of the capillaries in gastric mucosa, thus the gastric mucosal capillaries could be visualized. Having a high absorption of blue and green light by hemoglobin, NBI could enhance the vascular imaging [37]. A retrospective study analyzed the association between the ME-NBI findings and post-ER pathology in biopsy-proven gastric LGD lesions, the statistic results showed that 75% (48/64) of those lesions with positive ME-NBI findings (irregular microvascular or irregular microsurface) were gastric HGD or CA, while in those with negative ME-NBI findings, the updated diagnosis rate was only about 15% (11/73) [24]. The open diameter of a jumbo biopsy forceps is larger than that of conventional biopsy forceps, thus allowing an adequate amount of tissue to make a more reliable diagnosis [38]. A randomized trial compared the post-ER concordance rate between the conventional biopsy and the jumbo biopsy, it turned out that post-ER concordance rate in jumbo biopsy group was higher than that in conventional biopsy group, although the difference was not striking. Another study also reported that multisite biopsy would increase the diagnostic accuracy of EFB in gastric lesions significantly [21].

EMR allows the removal of large sessile or flat neoplasm, and ESD is an alternative technique enabling en bloc resection of large lesions [39]. Endoscopic resection has been widely used not only as a method of definitive treatment but also a diagnostic tool in gastrointestinal neoplasia in recent years [40]. Based on the results of this analysis, ER updated the diagnosis of EFB-proven LGD in a considerable proportion. As a result, it was suggested that if an EFB-proven gastric LGD lesion has all the above mentioned risk factors, ER should be performed so that the treatment of these patients wouldn’t be delayed [15,18].

The present review has several limitations. First, the analysis regarding UDR, HGD-UDR and CA-UDR were accompanied by high heterogeneities, but neither the regression analysis nor attempts to exclude potential outliers made it to reduce the heterogeneities. We believe that heterogeneity in diagnostic meta-analyses is common because of the observational nature of diagnostic studies [41]. Individual results varied among included studies, and outliers would affect the pooled values strikingly, which could explain the significant decreasement of the heterogeneity index by excluding 4 studies. In some situations, causes for between-study heterogeneity are difficult to elucidate [42]. In contrast to the meta-analysis of data from randomized controlled trials, substantial between-study heterogeneity is to be expected and must be incorporated in the models [43,44]. Second, most of the eligible studies were retrospectively designed, which might bring the errors associated with the retrospective retrieval of information. Third, we only selected articles written in English. Finally, the time interval between ER and EFB was available only in 4 studies, while not in the remaining 12 studies. This might be a source of heterogeneity, but subgroup analysis was not applicable due to a limited number of subjects. What’s worse, we couldn’t make sure the time interval was short enough so that the lesions wouldn’t progress. So further prospective, multi-center studies in which EFB-proven gastric LGD subjects will get ER within a short period of time are still needed.

In conclusion, this systematic review indicates that 25.0% of the EFB-proven gastric LGD lesions were diagnosed as advanced lesions (16.7% for HGD and 6.9% for CA) by ER. A size of 2 cm or greater, depressed morphology and nodular surface were among the most common risk factors indicating the discrepancy between EFB and ER pathologies.

## Supporting Information

S1 Checklist PRISMA Checklist. (DOC)

S1 Table Consequent *I* ^2^ after possible outliers were deleted for UDR. (DOC)

S2 Table Consequent *I* ^2^ after possible outliers were deleted for HGD-UDR. (DOC)

S3 Table Consequent *I* ^2^ after possible outliers were deleted for CA-UDR. (DOC)
