# Supplementary material for: How Commonly Is the Diagnosis of Gastric Low Grade Dysplasia Upgraded following Endoscopic Resection? A Meta-Analysis
Source: PLoS One. 2015 Jul 16;10(7):e0132699. doi: 10.1371/journal.pone.0132699 (PMC4504521; doi:10.1371/journal.pone.0132699)
Supplement: S1 Table — (DOC) [file pone.0132699.s002.doc]

**S1 Table. Consequent *I^2^* after possible** outliers were deleted for UDR

| Suriani,2011 (10.0%) |  | D | D | D | D |  |  | D |
| --- | --- | --- | --- | --- | --- | --- | --- | --- |
| Hwang,2012 (11.4%) |  |  | D | D | D |  |  |  |
| Lauwers,2004 (38.5%) |  |  |  |  | D |  | D | D |
| Tsuji,2012 (46.7%) |  |  |  | D | D | D | D | D |
| *I^2^* (%) | 88.9 | 89.1 | 88.8 | 86.0 | 87.0 | 86.4 | 87.2 | 87.5 |

UDR, upgraded diagnosis rate; D, deleted
