# Supplementary material for: How Commonly Is the Diagnosis of Gastric Low Grade Dysplasia Upgraded following Endoscopic Resection? A Meta-Analysis
Source: PLoS One. 2015 Jul 16;10(7):e0132699. doi: 10.1371/journal.pone.0132699 (PMC4504521; doi:10.1371/journal.pone.0132699)
Supplement: S3 Table — (DOC) [file pone.0132699.s004.doc]

**S3 Table. Consequent *I^2^* after possible outliers were deleted for CA-UD**R

| Park,2001 (6.7%) |  | D | D | D | D |  |  | D |
| --- | --- | --- | --- | --- | --- | --- | --- | --- |
| Kim,2006 (7.7%) |  |  | D | D | D |  |  |  |
| Lim,2014 (30.1%) |  |  |  |  | D |  | D | D |
| Won,2011 (30.8%) |  |  |  | D | D | D | D | D |
| *I^2^* (%) | 90.0 | 81.8 | 79.3 | 74.8 | 41.2 | 88.6 | 77.6 | 51.4 |

CA-UDR, carcinoma - upgraded diagnosis rate; D, deleted
